# Effect of Crown to Titanium Base Ratio and Force Angle on the Biomechanical Behavior of Dental Implants With CAD/CAM Zirconia and Lithium Disilicate Crowns: A Finite Element Analysis

**DOI:** 10.1002/cre2.70296

**Published:** 2026-02-09

**Authors:** Sara Abtahi, Shamim Mirzaboland, Allahyar Geramy, Marzieh Alikhasi, Hakimeh Siadat

**Affiliations:** ^1^ School of Engineering Faculty of Science and Engineering Macquarie University Sydney New South Wales Australia; ^2^ Department of Dental Biomaterials, School of Dentistry Tehran University of Medical Sciences Tehran Iran; ^3^ School of Dentistry Tehran University of Medical Sciences Tehran Iran; ^4^ Department of Orthodontics, School of Dentistry Tehran University of Medical Sciences Tehran Iran; ^5^ Department of Prosthodontics, School of Dentistry Tehran University of Medical Sciences Tehran Iran

**Keywords:** dental abutments, dental implant‐abutment design, finite element analysis, titanium base

## Abstract

**Objectives:**

This study investigates how titanium‐base (Ti‐base) abutment height, crown design, and force angulation affect biomechanics in an anterior single‐implant restoration, using finite element analysis.

**Material and Methods:**

A three‐dimensional anterior maxilla model was constructed with linear elastic properties. Two Ti‐base heights (3.5, 5.5 mm) and two crown heights (8, 11 mm) were tested as monolithic zirconia or bilayer (zirconia core veneered with lithium disilicate). A 146 N load was applied at the cingulum at 45° or 65°. von Mises stress (VMS) was computed in the crown, abutment, and surrounding bone.

**Results:**

A 3.5 mm Ti‐base with an 8 mm monolithic zirconia crown (SSZ) produced the lowest crown and abutment VMS. The highest crown VMS occurred in the 11 mm bilayer crown on a 5.5 mm Ti‐base (LLZEX) at 45°, while the highest abutment VMS occurred in the 3.5 mm Ti‐base with an 8 mm bilayer crown (SSZEX) at 65°. An increase in crown height raises crown stresses, whereas the impact of abutment height depends on configuration and angle. In bone, 45° loading increased VMS compared with 65° across all models.

**Conclusions:**

In anterior single‐implant models, the lowest restoration stresses were achieved with a short crown on a 3.5‐mm Ti‐base and monolithic zirconia. Long crowns (11 mm) increased crown stresses, and abutment height should be tailored to material and anticipated loading direction rather than adjusted by a single rule. Oblique loading consistently raised bone stress compared with 65°, underscoring the need to optimize for axial force transmission.

## 1 Introduction

Dental implant‐supported prostheses are the closest substitutes for natural teeth, providing a predictable and reliable therapeutic solution for missing teeth (Alam et al. 2015). Implant‐based restorations are now widely accepted by dental practitioners worldwide. Single‐tooth replacements, in particular, have a relatively high success rate, though there remains a possibility of failure in a small portion of patients (Jung et al. 2008). Early failures are primarily related to the surgical phase and occur due to biological reasons, leading to a deficient healing process in the peri‐implant tissue, while late failures can result from either biological or mechanical factors (Esposito et al. 1998a, 1998b; Mencio et al. 2017). Biologically, peri‐implant inflammation combined with occlusal overload can lead to progressive loss of osseointegration, accompanied by bone resorption (Masuelli et al. 2011; Chen et al. 2013).

Abutment height may influence peri‐implant tissue health, especially early marginal bone loss around bone‐level implants. Prior FEA studies examined abutment height in isolation and reported greater stresses with taller Ti‐bases under near‐axial loading (Beltrán‐Guijarro et al. 2024). The present study evaluates multiple interacting variables, including abutment height, crown height and material, and loading angle, to reflect the complexity of stress formation and distribution. Clinically, current evidence indicates that shorter abutments (≤ 2 mm) are associated with less early bone loss; a meta‐analysis by Chen et al. found approximately 0.52 mm less marginal bone loss at 6 months compared with abutments longer than 2 mm. Further research is needed to clarify the biomechanical consequences of abutment height on implants and peri‐implant tissues (Chen et al. 2019).

Recent advancements in digital‐processing technologies and the development of CAD/CAM systems, which play a crucial role in digital dentistry, have brought abundant benefits to implant‐based practices (Cardoso et al. 2020). This technology aids in determining the precise implant placement, enabling dentists to tailor abutments and crowns to each patient's unique needs (Tallarico 2020; Joda et al. 2017). CAD/CAM‐compatible titanium base abutments (Ti‐base) have been employed as a foundation for milling customized ceramic prostheses. Zirconia crowns are cemented onto the Ti‐base during chairside procedures, and this hybrid retention Ti‐base concept offers several benefits, including enhanced esthetics, improved reproduction of soft tissue contours, retrievability, and enhanced biomechanical performance (Silva et al. 2018).

A review article demonstrated that Ti‐base abutments exhibit excellent fracture resistance and longevity, especially when combined with resin cement, while also ensuring a proper marginal and internal fit (Al‐Thobity 2022). It should be noted that the clinical success and longevity of implant‐based restorations are largely influenced by biomechanical factors, including the type and magnitude of force, the surface structure and geometry of the implant, the quality and quantity of the surrounding bone, and the stress distribution at the bone–implant interface (Alikhasi et al. 2014).

Previous studies have examined the mechanical characteristics of restorations with different applied materials, stress distribution in retention screws with varying crown‐to‐implant ratios and gingival heights, in Ti‐base, and in implants with different diameters and lengths (Jayakumar et al. 2021; Moraes et al. 2015; Hossein Nattaj Miandeh et al. 2019; Adolfi et al. 2020; Raaj et al. 2019; Nguyen et al. 2023; Poovarodom et al. 2023; Strazzi‐Sahyon et al. 2023). Additionally, mechanical factors such as fracture resistance in various abutments, the effect of stress on the implant on the surrounding bones, and the impact of different materials have also been investigated (Elsayed et al. 2017; Atsü et al. 2019; Moraes et al. 2013; Robau‐Porrua et al. 2020; Kaleli et al. 2018).

While previous studies have explored various aspects of mechanical characteristics in implant‐based restorations, the specific investigation of the crown‐to‐Ti‐base length ratio's impact on stress distribution remains less discussed. One study found that although a larger crown‐to‐implant ratio did not significantly affect crestal bone levels in single‐tooth locking‐taper implants, it was associated with increased prosthetic problems (Urdaneta et al. 2010). Conversely, another study reported that within a 5‐year period, the crown‐to‐implant ratio had no significant impact on the clinical performance of implants supporting single‐crown restorations in the posterior jaw regions (Schneider et al. 2012). A 3D finite element analysis indicated that shorter abutments resulted in increased average stress (Naveau et al. 2009). Another study observed that vertical loading caused least stress on the supporting bone, while changes in force angle increased stress on the surrounding bone (Sütpideler et al. 2004). Additionally, it was suggested that crown height space has a more substantial effect on marginal bone stress compared to high crown‐to‐implant ratios or implant lengths (da Rocha Ferreira et al. 2021).

**Figure 1:**
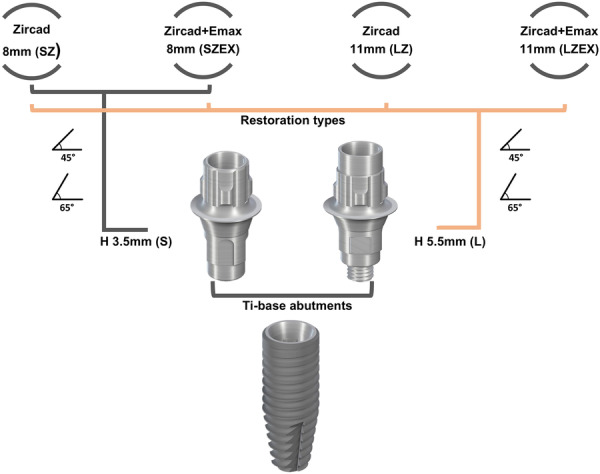
Graphical abstract, demonstrating study groups regarding abutment and crown heights, restorative ceramic materials, and force angle.

Given the critical role of biomechanical factors in treatment success and restoration longevity, this study aims to explore how variations in crown‐to‐abutment height ratios and force angles influence stress distribution in dental implants with different ceramic restorations (Figure 1). The null hypothesis being tested is that changes in crown‐to‐abutment height ratios, force angles, and material selection do not significantly affect stress distribution in these implant‐based restorations.

## 2 Material and Methods

### 2.1 3D Model Design of Maxilla

Based on the literature, a 3D model of the anterior maxilla was developed, which includes a thin, dense, and porous cortical bone layer and a fine trabecular layer with cancellous structure. The mechanical properties of the implant components, materials, and surrounding anatomy were specified within the software and are detailed in Table 1 (Alikhasi et al. 2014; Poovarodom et al. 2023; Siadat et al. 2015; Wazeh et al. 2018; Trindade et al. 2018) (Table 2).

**Table 1:** Material properties used in finite element model.

Material	Young's modulus (GPa)	Poisson's ratio
Cortical bone (Alikhasi et al. 2014)	34	0.26
Cancellous bone (Siadat et al. 2015)	13.4	0.38
Titanium alloy (Ti‐6Al‐4V) (Wazeh et al. 2018)	110	0.35
IPS e.max ZirCAD (Poovarodom et al. 2023)	200	0.35
IPS e.max CAD (Trindade et al. 2018)	95	0.22

**Table 2:** Maximum von mises stress (MPa).

Model	Force angle (degrees)	Crown VMS (MPa)	Abutment VMS (MPa)	Alveolar bone VMS (MPa)
SSZ	45	367.26	14.08	6.86
SSZ	65	482.43	32.20	3.85
SSZEX	45	418.44	34.26	4.92
SSZEX	65	585.66	38.76	3.16
LSZ	45	563.92	38.13	5.39
LSZ	65	617.31	28.81	2.84
LLZ	45	656.53	38.06	5.40
LLZ	65	645.48	26.30	2.87
LSZEX	45	453.87	32.14	5.31
LSZEX	65	445.79	26.16	2.80
LLZEX	45	663.19	31.36	5.05
LLZEX	65	657.29	30.93	2.77

*Note:* Stress values are reported as maximum von Mises stress (VMS) at each component.

Abbreviations: LS = long abutment and short crown, LL = long abutment and long crown, SS = short abutment and short crown, Z = zirconia crown, EX = zirconia + e.max layered crown.

### 2.2 Implant Design

Each designed model included a regular implant (RC implant CrossFit, Straumann, AG Waldenburg, Switzerland) with a length of 12 mm, a Ti‐Base (Variobase, Straumann, AG Waldenburg, Switzerland), a central maxillary ceramic crown, cortical bone, cancellous bone, and resin cement. The angle between the fixture and the frontal plane was set to 112^°^ to prevent the screw access hole from emerging from the buccal cortical layer (Musa and Ereifej 2023). The Ti‐Bases used in this study had two different lengths—short (3.5 mm) and long (5.5 mm)—both with a collar length of 1 millimeter. The study included the following groups:

SSZ: 3.5 mm abutment height (short) with an 8 mm (small) monolithic zirconia crown (IPS e.max ZirCAD LT, Ivoclar AG, Schaan, Liechtenstein).

SSZEX: 3.5 mm abutment height (short) with an 8 mm (small) bi‐layer zirconia core and veneering lithium disilicate crown (IPS e.max CAD, Ivoclar AG, Schaan, Liechtenstein).

LSZ: 5.5 mm abutment height (long) with an 8 mm (small) monolithic zirconia crown.

LLZ: 5.5 mm abutment height (long) with an 11 mm (large) monolithic zirconia crown.

LSZEX: 5.5 mm abutment height (long) with an 8 mm (small) bi‐layer zirconia core and veneering lithium disilicate crown.

LLZEX: 5.5 mm abutment height (long) with an 11 mm (large) bi‐layer zirconia core and veneering lithium disilicate crown.

All bilayer crowns were modeled as two distinct layers with separate material definitions: a zirconia core and a lithium disilicate veneer. The type of cement used in all models was Kuraray‐Panavia SA (Kuraray Noritake Dental, Japan) Luting Plus dual‐polymerized resin cement. According to the study of Kaleli et al. the Poisson's ratio and Young's modulus for this type of cement were considered equal to 0.28 and 18.6 (GPa), respectively (Kaleli et al. 2018). This type of cement was chosen due to its higher fracture resistance than non‐adhesive types (Conrad et al. 2007). The thickness of resin cement in all models was 140 μm (Wadhwani et al. 2010).

### 2.3 Force Application

A force of 146 Newtons, representing the maximum anterior bite force, was applied to the cingulum of all samples at two different angles—45^°^ and 65^°^—relative to the longitudinal axis of the maxillary central tooth. The 65^°^ angle is closer to the tooth's axis, while the 45^°^ angle is more off‐axis. In individuals without parafunctional habits, the duration of this force on the maxillary anterior teeth is significantly shorter than the average chewing force, which is also much smaller in magnitude (Alikhasi et al. 2014).

### 2.4 Finite Element Analysis

All models were developed in 3‐MATIC software (Materialize) and exported as STL files. The STL files were then converted to STP/Parasolid format using GEOMAG software for import into ANSYS Workbench (ANSYS R15.0; ANSYS Inc) for finite element analysis.

**Figure 2:**
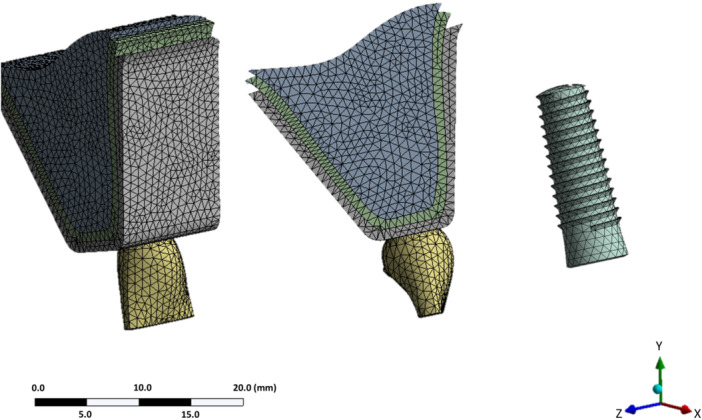
The meshing of components using ANSYS software.

Each model was discretized into approximately 136,000 nodes and 84,000 elements (Figure 2). The implant–abutment interface was defined as a bonded contact, assuming complete fixation. The abutment screw preload was simulated by calculating the torque force using the equation T = KDP and applying it as prestress in the first step of analysis.

To simulate functional loading, a vertical force of 146 N, representing the maximum anterior bite force, was applied at the cingulum of the crown at two angulations: 45° (oblique to the long axis) and 65° (closer to the long axis). Following simulation, von Mises stress was calculated for the crown, abutment, and surrounding cortical and cancellous bone to evaluate stress distribution and potential risk of material failure under different loading conditions.

## 3 Results

### 3.1 Stress Distribution in Ceramic Restoration

**Figure 3:**
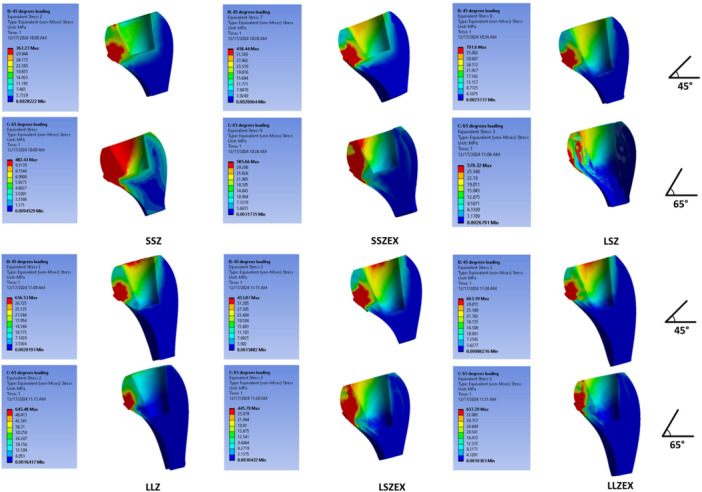
Stress distribution in ceramic restorations under 45° (top) and 65° force (bottom).

At the crown level, the highest VMS at 45° was observed in LLZEX (663.19 MPa), followed by LLZ at 45° (656.53 MPa); at 65°, the same ranking held (LLZEX 657.29 MPa > LLZ 645.48 MPa). For 8 mm crowns, increasing abutment height from 3.5 to 5.5 mm increased crown VMS in monolithic zirconia (SSZ → LSZ: 367.26 → 563.92 MPa at 45°; 482.43 → 617.31 MPa at 65°). In bilayer (EX) crowns, this abutment‐height effect was angle‐dependent crown VMS increased from SSZEX to LSZEX at 45° (418.44 → 453.87 MPa) but decreased at 65° (585.66 → 445.79 MPa). Force‐angle effects were model dependent, LLZ/LLZEX showed higher crown VMS at 45° than 65°, whereas SSZ and SSZEX showed the opposite (e.g., SSZ: 367.26 vs. 482.43 MPa; SSZEX: 418.44 vs. 585.66 MPa). Material comparisons at matched geometry indicated that the EX crown exceeded monolithic zirconia in SSZ vs. SSZEX and LLZ vs. LLZEX at both angles, whereas LSZEX was lower than LSZ at both angles (Figure 3).

### 3.2 Stress Distribution in Abutments

**Figure 4:**
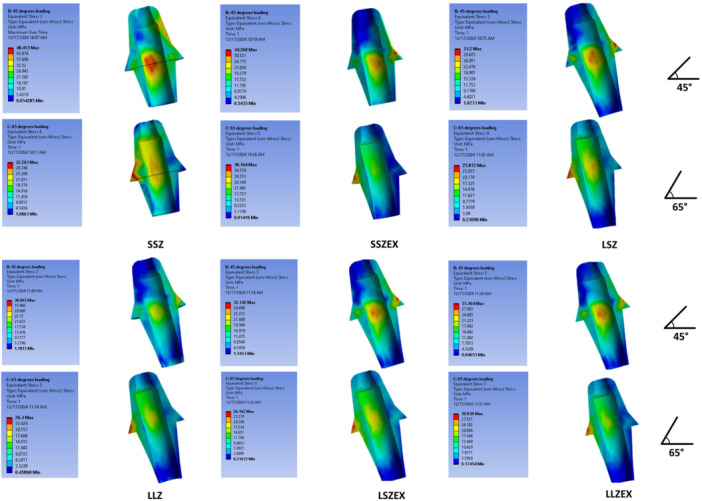
Stress distribution in the abutments under 45° (top) and 65° force (bottom).

At the abutment level, the numerically highest VMS was observed in SSZEX at 65° (38.76 MPa), followed closely by LSZ at 45° (38.13 MPa) and LLZ at 45° (38.06 MPa); given the small decimal differences and absence of inferential statistics, these distinctions should be interpreted as trends rather than definitive effects. In bilayer crowns, the shorter abutment (3.5 mm) showed higher abutment VMS numerically than the longer abutment (5.5 mm) at both angles (SSZEX vs. LSZEX: 34.26 vs. 32.14 MPa at 45°; 38.76 vs. 26.16 MPa at 65°). In monolithic zirconia, the pattern depended on angle: at 65°, the shorter abutment was higher (SSZ 32.20 MPa vs. LSZ 28.81 MPa), whereas at 45° the longer abutment was higher (LSZ 38.13 MPa vs. SSZ 14.08 MPa). For 11 mm crowns, LLZ exceeded LLZEX at 45° (38.06 vs. 31.36 MPa), while LLZEX exceeded LLZ at 65° (30.93 vs. 26.30 MPa). Overall, angle effects were configuration‐dependent, LSZ and LLZ abutment stresses were higher at 45° than 65°, whereas SSZ and SSZEX were higher at 65° than 45° (Figure 4).

### 3.3 Stress Distribution in Alveolar Bone

**Figure 5:**
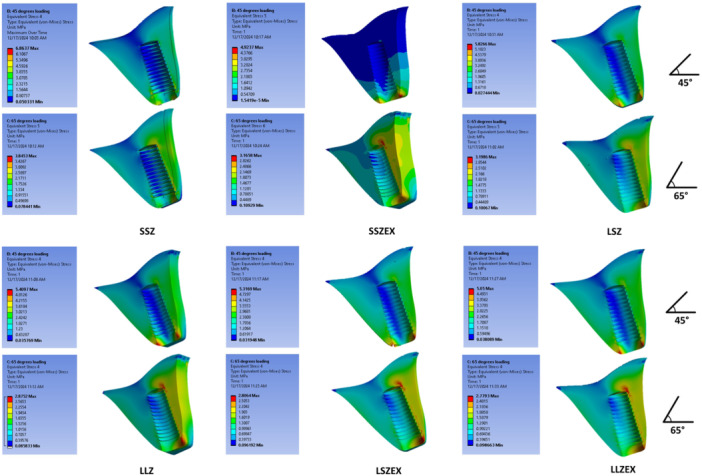
Stress distribution in the surrounding bone under 45°‐ (top) and 65°‐ force (bottom).

At the bone level, 45° loading consistently produced higher VMS than 65° across all configurations. The numerically highest bone VMS was observed in SSZ at 45° (6.86 MPa), while the lowest occurred in LLZEX at 65° (2.77 MPa). Variations attributable to abutment height, crown height, or material were modest relative to the effect of loading angle. Bone stresses were consistently far lower than those in the crown and abutment across all six configurations (Figure 5).

## 4 Discussion

One of the most significant challenges faced by dentists is reconstructing edentulous areas and replacing missing teeth. Permanent tooth loss can occur due to various factors, including caries, trauma, or unsuccessful treatments. To date, the most effective approach in such cases is implant‐based restoration (Siadat et al. 2015).

This study evaluated how crown height and Ti‐base height influence stress distribution. Crown height increases generally raised crown VMS, whereas the effect of abutment height on crown and abutment VMS was configuration‐dependent rather than uniform. Unlike a prior FEA that isolated abutment height and reported a near‐linear rise in transferred stress with taller Ti‐bases under near‐axial loading, our design varied multiple factors simultaneously (Beltrán‐Guijarro et al. 2024). In this multifactor context, abutment‐height effects were configuration‐ and angle‐dependent, indicating the relationship cannot be reduced to a single‐factor rule. Clinically, this argues for case‐specific abutment selection rather than a uniform “longer vs. shorter” recommendation.

The lowest crown VMS occurred in SSZ, and the highest in LLZEX at 45°. These findings are consistent with a vertical‐cantilever effect, whereby increased crown height elevates crown stresses.

Angle effects were model‐dependent at the crown, LLZ and LLZEX showed higher crown VMS at 45° than 65°, whereas SSZ and SSZEX showed the opposite trend. This aligns with the notion that oblique forces are more off‐axis but also indicates that internal geometry and material interact with angulation. Prior finite element analysis and clinical studies have linked larger crown height ratios with increased prosthetic complications such as screw loosening, breaking of implant components, and porcelain chipping, but not necessarily with adverse bone outcomes (Moraes et al. 2015; Hossein Nattaj Miandeh et al. 2019; Urdaneta et al. 2010).

Considering that the angle of 65° is closer to the longitudinal axis of the tooth, and mastication tends to be more vertically directed, our observation that oblique (45°) loading produces higher stresses contrasts with the findings of Schneider et al. who reported that the crown‐to‐implant length ratio did not affect marginal bone levels or overall clinical performance. Their analysis emphasized posterior implants and clinical endpoints, whereas the present work addresses an anterior scenario using FEA under static loading; differences in implant location, loading direction, and outcome measures likely account for the divergent results Schneider et al. 2012.

Jayakumar et al. examined chrome–cobalt abutments of 5.5, 3.5, and 3.6 mm under vertical (36 and 100 N) and 30° loading; the 3.6 mm abutment under 36 N vertical force showed favorable deformation and shear‐stress behavior (Jayakumar et al. 2021). In our models, abutment height did influence VMS, but the effect was material‐ and angle‐dependent. The maximum crown VMS occurred in LLZEX at 45°, whereas the maximum abutment VMS occurred in SSZEX at 65°. For 8 mm monolithic crowns, increasing abutment height increased crown VMS at both angles. In bilayer crowns, increasing abutment height increased crown VMS at 45° but decreased it at 65°.

At matched geometry, material effects were not uniform, SSZEX exceeded SSZ and LLZEX exceeded LLZ at both angles, whereas LSZEX was lower than LSZ at both angles.

For bilayer crowns, the shorter abutment produced higher abutment VMS at both angles. For monolithic crowns, the effect was angle‐dependent: the shorter abutment exceeded the longer at 65°, whereas the longer exceeded the shorter at 45°. While prior studies linked shorter abutments to greater stresses (Naveau et al. 2009) our data show this effect is context‐dependent, varying with force angulation and material configuration rather than holding uniformly.

At the bone level, loading angle predominated (45° > 65° across all models), while differences attributable to crown height, abutment height, or material were modest. Nattaj et al stated that increasing the vertical height of the crown reduces the tension of the implant screw and increases the tension of the abutment and fixture. It also increases the compressive and tensile stress in the surrounding bone structure (Hossein Nattaj Miandeh et al. 2019). In a study conducted by Sütpideler et al. using the finite element analysis method, it was stated that the change in the angle of the applied force leads to the introduction of stresses with larger values to the bone surrounding the implant (Sütpideler et al. 2004). Similarly, in the present study, applying force at an angle of 45° in all models caused more VMS in the implant than at 65°. Findings from Moras et al. further support greater stress concentration and displacement with increased crown height, particularly under 45° loading. Moraes et al. (2013); Robau‐Porrua et al. (2020).

Based on the observed numerical differences in von Mises stress distributions between models of varying abutment height, crown length, and material combinations, the null hypothesis is rejected.

## 5 Conclusions

Within the limitations of this finite element study, a shorter crown on a 3.5 mm Ti‐base with a monolithic zirconia crown (SSZ) produced the lowest crown and abutment stresses among the tested designs on anterior single‐implant models. Longer crowns (11 mm) increased crown stresses, and the impact of abutment height on crown and abutment stress was configuration and angle‐dependent. At bone level, oblique 45° loading consistently exceeded 65°, underscoring the need to favor axial load transfer.

It should be noted that this study assumed fixed boundary conditions and a simplified model compared with the biological situation. To confirm these findings, future work incorporating fatigue loading, biological variability, and experimental validation is recommended to establish their clinical relevance.

## Author Contributions

**Sara Abtahi:** formal analysis, data curation, visualization, methodology, writing – original draft preparation, writing – review and editing. **Shamim Mirzaboland:** investigation, data curation, validation, writing – original draft preparation. **Allahyar Geramy:** methodology, validation, writing – review and editing. **Marzieh Alikhasi:** conceptualization, resources, supervision, writing – review and editing. **Hakimeh Siadat:** conceptualization, supervision, funding acquisition, project administration, methodology, writing – review and editing.

## Ethics Statement

Ethical approval for this study was granted by the ethics committee of Tehran University of Medical Sciences (IR.TUMS.DENTISTRY.REC.1400.096).

## Conflicts of Interest

The authors declare no conflicts of interest.

## Data Availability

The data that support the findings of this study are available from the corresponding author upon reasonable request.
